# Willingness To Pay for Social Health Insurance in Iran

**DOI:** 10.5539/gjhs.v6n5p154

**Published:** 2014-05-30

**Authors:** Shirin Nosratnejad, Arash Rashidian, Mohsen Mehrara, Ali Akbari Sari, Ghadir Mahdavi, Maryam Moeini

**Affiliations:** 1 School of Public Health, Tehran University of Medical Sciences, Tehran, Iran; 2 Knowledge Utilization Research Center, Tehran University of Medical Sciences, Tehran, Iran; 3 School of Economics, Tehran University, Tehran, Iran; 4 ECO College of Insurance, Allameh Tabataba’i University, Tehran, Iran

**Keywords:** Willingness To Pay, health insurance, Iran, contingent valuation method

## Abstract

**Objective:**

The substantial level of out-of-pocket expenditure for health care by the population causes policy makers to draw particular attention to the proposal of a social health insurance for uninsured members of the community. Hence, it is essential to gather reliable information about the amount of Willingness To Pay (WTP) for health insurance. We assessed the WTP for health insurance in Iran in order to suggest an affordable social health insurance.

**Method:**

The study sample included 300 household heads in all Iranian provinces. The double bounded dichotomous choice approach was used to elicit the WTP.

**Result:**

The average WTP for social health insurance per person per month was 137 000 Rial (5.5 $US). Household heads with higher levels of education, income and those who worked had more WTP for the health insurance. Besides, the WTP increased in direct proportion to the number of insured members of each household and in inverse proportion to the family size.

**Conclusions:**

From a policy point of view, the WTP value can be used as a premium in a society. An important finding of this study is that although households’ Willingness To Pay is not more than the total insurance premium, households are willing to pay more than the premium they ought to pay for health insurance coverage. That is, total insurance premium is 150 000 Rials and households ought to pay approximately half of this sum. This can afford policy makers the ideal opportunity to provide good insurance coverage for medical services according to the need of society.

## 1. Introduction

Many low and middle income countries face challenges regarding the financing of health care, especially since limited taxation policies and the complex management of health insurance are major barriers that cause inadequate health expenditure per capita in these countries (Vialle-Valentin, Degnan, & Ntaganira, 2008).

Insufficient health financing can result in excessive out-of-pocket spending for health care by the population. This out of pocket spending not only limits the utilization of health care, but it also indirectly affects the health and productivity of people (Dong, Kouyate, Cairns, Mugisha, & Malin, 2003). High out of pocket expenditure leads to policy makers drawing particular attention to the proposal of health insurance for the uninsured members of the community.

In 1994, Iran’s Parliament introduced legislation on the Universal Health Insurance. But, according to the latest reports, 16.8% of the Iranian population are not supported by any health care insurance (Rashidian et al., 2010) and about 50% of total health care expenditure in the country is paid directly by the households in the form of out-of-pocket expenditure (Manenti, 2011; Takian, Rashidian, & Kabir, 2011).

In this country, mandatory social health insurance is limited to certain groups of the Iranian population and others have to demand voluntary health insurance. Many suggest that the primary factor hindering the access of households to health insurance in countries, in which people are familiar with the concept of health insurance coverage, is that insurance coverage is simply not affordable (Bundrof & Pauly, 2006). In most countries, health insurance providers have tried to develop various strategies that make health insurance more affordable (Bundrof & Pauly, 2006). Hence, it is essential to gather reliable information about the amount that uninsured people are willing to pay for health insurance coverage. It is also important to determine the major factors that affect people’s choice of payment for health insurance.

In addition, the households’ contribution to the insurance is considered as a useful tool which can provide uninsured households with an opportunity to receive good insurance coverage especially in low and middle income countries.

The purpose of this paper was to estimate the Willingness To Pay (WTP) for social health insurance among uninsured households in Iran, applying a Contingent Valuation Method (CVM) to suggest an affordable health insurance plan for uninsured households. Moreover, we analyzed the effective variables that were associated with the WTP of Iranian households.

## 2. Method

### 2.1 Study Site

Iran which is a middle- income country with a population of more than 75 million in 2012, spends about 6.3% of its GDP on health services (WHO, 2013). The health services in Iran are generally divided into two categories of: 1-Primary Health Care (PHC), that is fully financed by the government, and is delivered almost free of charge to all residents of the country, and 2- treatment services that are offered by over 1000 hospitals (mostly public hospitals) and many private ambulatory service outlets including pharmacies, physicians’ offices, diagnostic services etc. The cost of these treatment services is usually incurred by patients, which can be substantial if they aren’t insured, and especially if the services are offered by the private sector (in press, 2014).

The latest survey in Iran, carried out in 2010 found that 16% of urban people don’t have any health insurance coverage (Rashidian et al., 2010). The health insurance coverage in this country is linked to employment. While employed people are usually covered by the social health insurance services, most uninsured people are those that work mostly in the informal sector, have short term contracts with the public or private sectors, or are self employed or unemployed and reside in cities or big towns (rural residents are under compulsory coverage which is offered almost free of charge).

### 2.2 Choice of WTP Method

The Contingent valuations (CV) are survey methods for eliciting the WTP for non-market goods. In these methods, first, the good and hypothetical market is described to the respondents and then, they are asked to state the maximum amount which they are willing to pay.

There are four methods for CV studies: open ended questions, bidding game, payment card, dichotomous choice method (single or double bounded) (Venkatachalam, 2004).

Related studies applied different methods of CV for estimating the WTP for health insurance in developing counties. For example, Ghana 1992 (Asenso-Okyere et al., 1997), Burkina Faso 2001 (Dong, Kouyate, Cairns, Mugisha, & Malin, 2003), Iran 2001 (Asgary, Willis, Taghvaei, & Rafeian, 2004), Cameroon 2002 (Binam, Nkama, & Nkendah, 2004) and Nigeria 2007 (Onwujekwe et al., 2010) used the bidding game method, Nigeria 2007 (Ataguba, Ichoku, & Fonta, 2008), Cameroon 2009 (Donfouet, Essombè, Mahieu, & Malin, 2011a; Donfouet, Essombè, Mahieu, & Malin, 2011b) and India 2010 (Ghosh & Mondal, 2011) used the double bounded Dichotomous choice method and China 2000 (Barnighausen, Liu, Zhang, & Sauerborn, 2007), used payment card for estimating the WTP for health insurance.

Each method has some advantages and disadvantages. In this paper, we applied the double-bounded dichotomous choices (DBDC) (also known as ‘referendum format’) to estimate the WTP for health insurance in Iran. In dichotomous choice methods, the respondent only answers ‘yes’ or ‘no’ to a given two questions about the WTP amount. The first question is followed by another question specifying a lower amount, if the answer to the first question were negative, and a higher amount, if it were positive (Venkatachalam, 2004). To avoid the initial bid bias, we used four different starting bids.

In this method, the respondents’ answers were divided into four groups: “yes, yes”, “yes, no”, “no, yes” and “no, no”. Comparing this method with other elicitation methods, this procedure has been found to have the most significant statistical efficiency (Ghosh & Mondal, 2011; Lang, 2010).

### 2.3 Sample Size and Data Collection

According to the 2010 Iran multiple- indicator Demographic and Health Survey, 84% of Iranian households were supported by health insurance (Rashidian et al., 2010). Therefore, given that the probability of having health insurance by an Iranian household was 84%, and by a confidence interval of 95% and a power of test of 80%, the sample size of the study should include 285 households, but since the study was a cross sectional one, we considered a sample size of 300 households in order to avoid the sample attrition resulting from the questionnaires that had not been responded to.


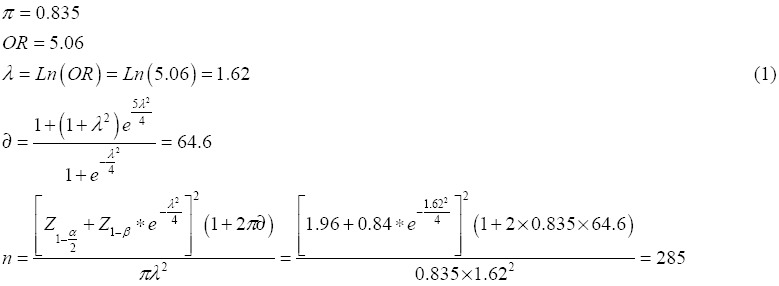


This was a cross sectional study and a questionnaire survey was administrated to the main study sample including 300 urban households in all Iranian provinces randomly chosen from among the 5000 households without any health insurance coverage, as indicated by the 2010 Iran multiple- indicator Demographic and Health Survey (Rashidian et al., 2010). The data collection method was telephone interview with household heads. The heads of the selected households were interviewed in November 2013 by two trained interviewers who were students of public health.

### 2.4 Questionnaire

The questionnaire, which was designed according to the guideline of the Contingent Valuation (CV) studies (Venkatachalam, 2004), was a structured questionnaire. Validity and reliability of the CV questionnaire has been confirmed repeatedly (Lang, 2010; Klose, 1999). The questionnaire consisted of four parts. In the first part, the interviewers described the purpose of the study and asked each respondent whether or not they had health care insurance coverage. In the following part, the hypothetical scenarios of the WTP for the health insurance were described to the respondents. The bids randomly assigned to each household head were 50 000, 100 000, 150 000 and 200 000 Rials, in order to avoid starting point bias (lower and upper bids were determined by open- ended question performed on the pilot sample). In the third part, each household head was asked about the health status of them and their family members, and the final part included the socio-demographic questions. Neither respondents’ names nor other characteristics were identified after interviews were recorded. We received ethical approval for conducting this study.

### 2.5 Econometric Model

The method known as the double- bounded or interval data model allows the efficient use of data in order to estimate the WTP. As mentioned above, in this method, we have four kinds of responses to the WTP questions: (A) ‘yes’ – ‘no’, (B) ‘yes’ – ‘yes’, (C) ‘no’ – ‘yes’, (D) ‘no’ – ‘no’.

The function to be estimated is:


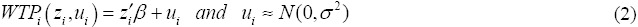


Afterwards, the probability of each of the four cases is defined as:


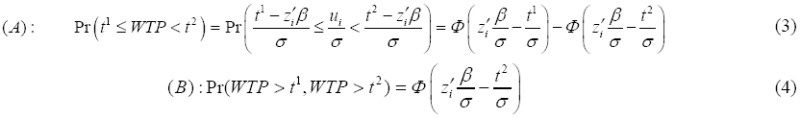



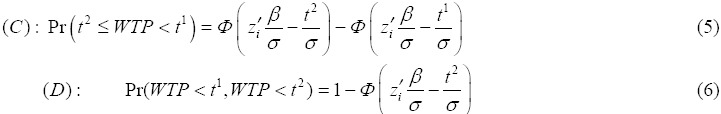


Estimation of *β* and *σ* was based on maximum likelihood method. The function that needs to be maximized to find the parameters of the model is:


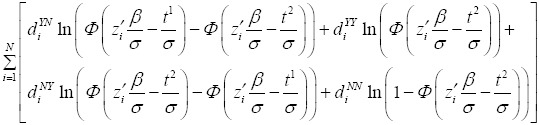


Where 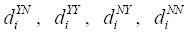 are indicator variables that take the value of one or zero depending on the relevant case for each individual. Each household head contributes to the logarithm of the likelihood function in only one of its four parts. Here, we obtain directly β and σ then we can estimate WTP (Lopez-Feldman, 2012).

## 3. Result

The characteristics of the sample are presented in Table 1.

**Table 1:** The characteristics of the households in the sample

Variable name	Description	Mean or proportion	Std. Dev
Gender	Indicated the gender of each household head: 1 for a male, 0 for a female	0.93	0.26
Family size	Indicated the number of people in each family (continuous)	3.81	1.34
Experience	To Describe previous use of health insurance: 1 if insurance had been used in the past, 0 if not	0.45	0.03
No. insured	Indicated the number of insured members in each family	0.27	0.71
Excellent health	Indicated the health status of each household head(a self report variable): 1 if health status were excellent, 0 if otherwise	base	-
Good health	Indicated the health status of each household head(a self report variable), 1 if health status were good, 0 if otherwise	0.78	0.024
Middle health	Indicated the health status of each household head(a self report variable), 1 if health status were medium, 0 if otherwise	0.17	0.022
Bad health	Indicated the health status of each household head(a self report variable), 1 if health status were bad, 0 if otherwise	0.05	0.134
Age	Indicated the age of each household head	46.51	12.80
Education	Indicated the education years of each household head	5.78	2.40
Past inpatient	Indicated the family’s utilization of inpatient services in the past: 1 if services had been used, 0 if not	0.20	0.024
Future inpatient	Indicated the family’s utilization of inpatient services in the future : 1 if they would be utilized, 0 if not	0.18	0.022
Drug –user	Indicated the number of family members using any drugs regularly	0.37	0.60
Disable	Indicated the number of disabled people in each family	.0270	0.01
Patient	Indicated the number of patients in each family	0.07	.017 0
Under5	Indicated the number of children under 5 years of age in each family	0.26	0.49
Over 65	Indicated the number of elderly people (over 65 years) in each family	0.15	0.44
Marriage	Indicated the marriage status of each household head: 1if married, 0 if otherwise	0.92	0.016
employment	Indicated the employment status of each household head: 1 if employed, 0 if otherwise	0.83	0.022
Retired	Indicated if each household head were retired, 1 if retired, 0 if not	0.15	0.021

Out of 300 administrated questionnaires, 290 were suitably completed and the response rate of the household heads was 87%. The mean number of family members was 3.81, ranging from 1 to 11. On average, the household heads were 46.51 years old, the average education degree of the household heads was middle school, 93% of household heads were male and 7% of them were female. 92% of household heads were married and 8% of them had never married, been widowed or separated. 83% of household’s heads were employed, 2% were unemployment and 15% were retired. Other characteristics of households are indicated in Table 1.

**Figure 1:**
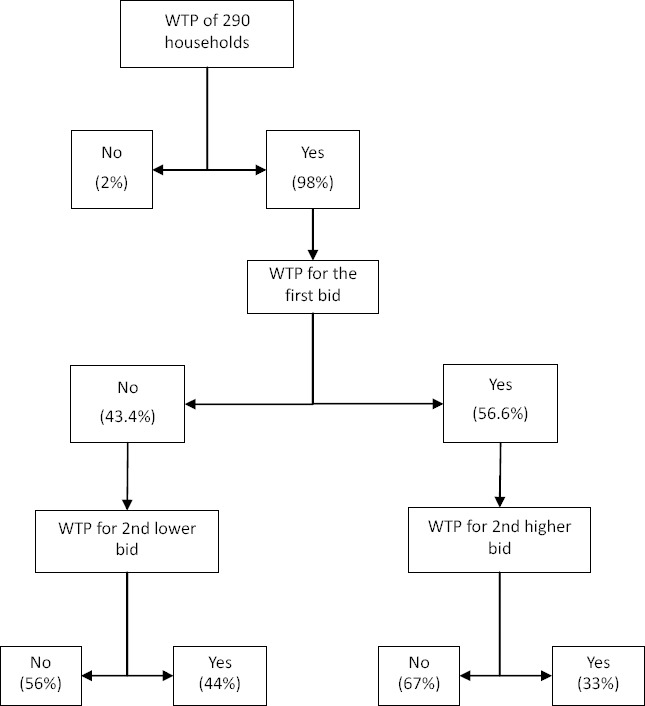
Summary of statistics of the responses to double bounded dichotomous choice question

Out of 290 households, 286 were willing to join the health insurance scheme. 56.6% of household heads responded “yes” to the first bid and 43.4% responded “No”. Figure 1 shows the summary of statistics of the responses to the double bonded dichotomous choice question.

**Figure 2:**
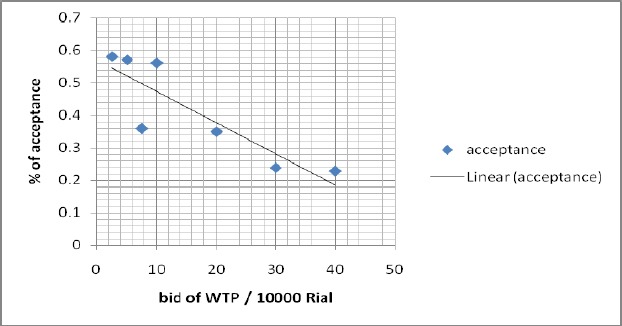
Household acceptance rate (%) and bids (10 000 Rial) (using double bounded dichotomous choice method)

Figure 2 shows the relationship between the probabilities of accepting different bids. The downward sloping graph shows an inverse relationship between price and acceptance rate and indicates that the probability of accepting decreased by increasing the bids. The probability of accepting the bids ranged from 58% for the lowest bid to 23% for the highest bid.

The results of the interval regression performed using Stata version 11 indicated that there was a significant relationship between the WTP of household heads and the demographic and their socio-economic characteristics.

Table 2 presents the estimated effects of the characteristics of household heads on the WTP for social health insurance.

**Table 2:** The effect of explanatory variables on the Willingness To Pay of household heads

Log likelihood = -357.64			Number of observation = 281			
			Wald chi2(20) = 73.66			
			P –value = 0.0000			
Variables	Coefficient	Standard Error	Z	P > |Z|	Confidence interval 95%	
Gender	7.91	5.47	1.45	0.148	-2.80	18.63
Past – inpatient	2.16	1.83	1.18	0.238	-1.43	5.75
Future – inpatient	2.30	2.02	1.13	0.257	-1.67	6.27
under5	.84	1.84	0.46	0.648	-2.77	4.45
over65	1.64	2.85	0.58	0.564	-3.94	7.23
Marriage	-9.25	5.35	-1.73	0.084	-19.74	1.23
Income	3.89	1.11	3.50	0.000	1.71	6.07
Education	1.54	0.75	2.06	0.039	0.07	3.02
Insured members	3.68	1.13	3.26	0.001	1.47	5.89
Drug- users	2.53	1.82	1.39	0.165	-1.04	6.11
Disable members	2.28	4.73	0.48	0.629	-6.99	11.55
Patients	.09	3.02	0.03	0.975	-5.83	6.02
Age	0.06	0.08	0.74	0.458	-0 .10	0.22
Family size	-1.63	0.61	-2.65	0.008	-2.84	-0.42
Experience	1.74	1.52	1.15	0.251	-1.23	4.72
Employed	6.88	2.76	2.49	0.013	1.47	12.29
Retirement	7.26	5.70	1.27	0.203	-3.92	18.44
Good health	-0.86	1.86	-0.47	0.642	-4.50	2.78
Middle health	-3.78	2.5	-1.48	0.138	-8.78	1.21
Bad health	-6.27	4.1	-1.52	0.128	-14.34	1.80
constant	-0.64	5.71	-0.11	0.910	-11.84	10.55
Sigma						
Constant	10.73	0.69	15.59	0.000	9.38	12.08

The results indicated that household heads with high education and income who were employed had more WTP for the health insurance. By increasing the insured members in each family, the WTP increased and by increasing family size, the WTP decreased.

The estimation of the WTP is demonstrated in Table 3.

**Table 3:** Mean Willingness To Pay of household heads per family member per month

	Mean	Standard. Error	P-value	Confidence interval 95%
**WTP (10 000 Rials)**	13.700	0.71	o.ooo	10.305 - 15.103
1 $US = 25 000 Rials				

The findings showed that the average of the WTP for the social health insurance per person per month was 137 000 Rials (5.5$) with a confidence interval of 123 050 to 151 030 (Rials). Therefore, this price was statistically significant.

## 4. Discussion

The findings of the paper demonstrated that mean amount that uninsured urban households in Iran are willing to pay for the social health insurance is monthly 137 000 Rials (5.5$) per person with confidence interval of 123 050 to 151 030 Rials. Therefore, considering the GDP per capita of Iran in 2012, we estimated the WTP of household heads to be around 1.39% of GDP per capita.

We also examined the effect of socio-economic and health statues variables on the WTP of households. Our findings suggested that education, family size, and the number of insured family members and income are significant variables of the WTP, but the variables of health status aren’t significant on the WTP of household heads.

The positive coefficient of education indicated that educated household heads are more willing to pay than others. Earlier studies have shown this relation between education and the WTP for health insurance (Tsai-Ching & Chin-Shyan, 2002; Dong, Kouyate, Cairns, Mugisha, & Sauerborn, 2003; Ataguba, Ichoku, & Fonta, 2008; Lofgren, Thanh, Chuc, Emmelin, & Lindholm, 2008; Donfouet, Essombè, Mahieu, & Malin, 2011a) as observed in this study. The coefficient of family size is negative and statistics imply that by increasing family size, the WTP of the household decreased. In addition, it is rational that the heads of larger households have to pay more premiums, which may result in less WTP for insurance coverage. This result has also been supported by earlier works (Dror, Radermacher, & Koren, 2007; Asenso-Okyere et al., 1997; Mathiyazhagan, 1998; Binam, Nkama, & Nkendah, 2004; Lofgren, Thanh, Chuc, & Emmelin, 2008) and the coefficient of insured members of each family is significant and positive, which is indicated by the fact that increasing numbers of insured family members led to an increase in that household’s WTP.

The variable of employment status is significant indicating that household heads that were employed had more WTP for the health insurance. This finding is well established in the literature (Mathiyazhagan, 1998), because employed people have regular income and health insurance is affordable for them, in comparison with the unemployed.

An important variable in the decision of households to pay for social health insurance scheme is income. The positive coefficient of income is in conformity with economic theory. In fact, according to theory, there must be a positive relationship between income and the WTP. Income has a positive and statistically significant impact on the WTP. This seems to be in agreement with findings by other researchers showing that income has a positive and significant influence on the WTP for social health insurance meaning that social health insurance schemes are normal goods (Mathiyazhagan, 1998; Dong, Mugisha, Gbangou, Kouyate, & Sauerborn, 2004; Barnighausen, Liu, Zhang, & Sauerbom, 2007; Chen, Lee, Hung, & Lin, 2008).

The results indicate that the most significant and effective variables on the WTP of household are socio-economic and demographic variables that show the macro economic and cultural status of country. Then again, these variables are difficult to manipulate by policy makers.

The other study that conducted among rural households in Iran in 2001 yielded 2.77 US$ per household per month (when social health insurance was not free for them). This amount is equal to 1.90% of GDP per capita (Asgary, Willis, Taghvaei, & Rafeian, 2004).

Comparing the WTP of the last study and the WTP of our study indicated that the nominal amounts of the two studies are different, but when we calculated the amounts as a GDP per capita the WTP of last study is more than of the WTP of our study.

The latest systematic review of the WTP for health insurance in low and middle income countries showed that the average of the WTP of individuals was 1.25% (0.37–2.13%) of GDP per capita, and 1.74% (0.71–2.78%) when adjusted for the net national income per capita in these countries (in press, 2013). Our study calculated this amount, 1.39% of GDP per capita, they are nearly equal.

From a policy point of view, the WTP value can be used as a premium in the society. In Iran, premium for social health insurance for self insured people is 150 000 Rials. But, self insured people that buy social health insurance can voluntarily share the premium with the government; therefore, self insured people pay only 50% of the premium, which is equal to 75 000 Rials each month. It is indicated that Iranian households are willing to pay more than the premium for the health insurance coverage. This can provide policy makers with the ideal opportunity to provide good insurance coverage for medical services, according to the needs of society in order to extend better and more sufficient medical services.

The result of CVM indicated the high health care expenditure in society. And also the findings indicated the monetary value of health insurance. They were also applied as a cost-benefit analysis to show the benefit of health insurance in comparison with its cost.

The results also proved that there was no adverse selection in the health insurance market of Iran, because we found no relation between the WTP and utilization of inpatient services in the past or expected use of them in the future, or having patients or disabled or regular drug user members in the family.

In this study we tried to use appropriate modeling approaches and test their underlying assumptions. We selected a specific sub-sample of households that did not have any coverage of health insurance and used a double bonded dichotomous choice format to examine the WTP of the Iranian households. The double bounded dichotomous choice method has significant statistical efficiency in comparison with other contingent valuation methods (Ghosh & Mondal, 2011; Lang, 2010). To avoid initial bid bias, which is common in the Contingent valuation method, we used four initial bids. An inverse relationship between prices and acceptance of health insurance confirmed that the health insurance schemes are normal goods in Iran. Besides, this important finding proved the construct validity or interval validity of this study (Venkatachalam, 2004).

There were some weaknesses in this study. First, finding uninsured household heads by telephone resulted in some difficulties. From the 717 households that we called, 376 phone numbers were not valid or no one answered them after three times dialing, and 44 households did not participate in our survey. This situation indicated that the sample of survey might not be a good representation of all uninsured households. Second, we considered the perceived quality of the services to be uniform for the entire study sample. Finally, the data coming from the questionnaire survey provided only a snapshot of the households’ behaviors. Long term prospective studies might provide better evidence for estimating the WTP of households and assessing the influence of different factors on the insurance purchase.

## 5. Conclusion

In conclusion, this paper set out to deliver the evidence on a maximal WTP among the uninsured households of Iran estimated to be 137 000 Rials (5.5$) or 1.39% of GDP per capita. Total insurance premium is 150 000 Rials and self insured people ought to pay approximately half of this sum, which is equal to 75 000 Rials each month. It is indicated that uninsured Iranian households are willing to pay more than the premium for the social health insurance. It can provide policy makers with the opportunity to provide good insurance coverage for the medical services in our society in order to offer better and more sufficient medical services.

The WTP for the health insurance positively correlated with income, education, employment statues of household heads and the number of insured members in each family. On the other hands, family size had a negative effect on WTP. It should be noted that these variables that significantly affected the WTP of the household heads are not easily influenced by the policy maker’s decisions. Altogether, the analysis suggests that unless nation-wide non-voluntary policies are implemented, it may be very difficult to substantially expand health insurance coverage.
